# Allergen-Specific Immunotherapy in Asthma

**DOI:** 10.1007/s40521-014-0013-1

**Published:** 2014-03-12

**Authors:** Marek Jutel

**Affiliations:** 1 Department of Clinical Immunology, Wrocław Medical University, Wroclaw, Poland; 2 ALL-MED Medical Research Institute, Wroclaw, Poland

**Keywords:** Asthma, Allergen-specific immunotherapy, SLIT, SCIT, Bronchodilators, Long acting bronchodilators, LABA, Inhaled corticosteroids, Omalizumab

## Abstract

Current asthma therapies can effectively control symptoms and the on-going inflammatory process; however, they do not affect the underlying, dysregulated immune response. Thus, they are limited to blunting the progression of the disease, which relapses on ceasing the treatment. Allergen-specific immunotherapy (AIT) is the only etiology-based treatment capable of disease modification. Recent evidence provided a plausible explanation for its multiple mechanisms inducing both rapid desensitization and long-term allergen-specific immune tolerance, as well as the suppression of allergic inflammation in the affected tissues. Although the current guideline documents give both subcutaneous (SCIT) and sublingual (SLIT) immunotherapy a conditional recommendation in allergic asthma due to the moderate and low quality of evidence, respectively, a growing body of evidence from double-blind, placebo-controlled studies shows that both SLIT and SCIT are effective in reducing symptom scores and medication use, improving quality of life, and inducing favorable changes in specific immunologic markers. Due to the very limited evidence from head-to-head comparative studies and variability of the end-point used in different studies, it is currently not possible to assess superiority of either route of vaccine administration.

## Introduction

Asthma is a chronic inflammatory disease with high incidence, about 300 million people worldwide. The pathological process of the airways is associated with hyperresponsiveness, which leads to recurrent episodes of wheezing, dyspnoea, chest tightness and cough, as well as variable airflow obstruction that may become permanent due to airway remodelling [1]. Asthma is not exclusively associated with allergy/atopy. However, more than 50 % of the asthmatic population is allergic/atopic, but only a fraction of allergic subjects develop asthma. Thus, the pathophysiology of asthma is very complex and includes several disease variants [2]. Distinct phenotypes of asthma describe clinical and morphologic characteristics as well as unique responses to treatment. In addition, various endotypes have been described that define intrinsically distinct pathogenetic mechanisms [2]. For a long time, asthma has been considered mainly a T_h_2 cell-mediated disorder with interleukin (IL)-4, IL-13, IL-9 and IL-5 involved in the airway inflammation [3]. However, many other cell types including Treg, T_h_1, T_h_17, natural killer (NK) and ββ T cells are also involved [2, 4, 5]. It is assumed that the more severe asthma symptoms develop, the more T_h_1 and T_h_17 cells are involved. In particular, neutrophilic infiltration and inflammation triggered by the production of tumor necrosis factor (TNF)β, IL-17, and IL-27 might account for corticosteroid resistance [6]. Thus, endotyping asthma based on disease mechanisms could eventually lead to individualized management.

Current asthma therapies can effectively control symptoms and the on-going inflammatory process; however, they do not affect the underlying, dysregulated immune response. Thus, they are limited to blunting the progression of the disease, which relapses on ceasing the treatment.

Bronchodilators are effective in reducing airway obstruction. Current guidelines recommend combined use of long-acting bronchodilators (LABA) with inhaled corticosteroids (ICS) due to safety concerns [7]. Alternative classes of bronchodilators, such as vasoactive intestinal peptide analogs and potassium-channel openers, are currently under investigation. ICS are currently basic controllers in asthma therapy. These ‘conventional’ anti-asthmatic agents are constantly updated both for the new molecules as well as more convenient and effective delivery devices, which also improve patient compliance. The major setbacks with the usage of ICS and LABA include the fear of long-term side effects, compliance with inhaled administration, as well as the relapse of symptoms after discontinuation of drug administration. In a considerable number of patients, acceptable symptom control is not achieved with these drug classes. Thus, considering the complexity of asthma pathogenesis, therapies aiming at blocking critical effector molecules are under intensive investigation [8]. Currently, omalizumab is the only anti-IgE monoclonal antibody approved for asthma treatment [9]. In addition, novel drugs utilizing immune-modulatory mechanisms including suppression of disease-associated cytokines are being developed (see this issue, Akdis M. et al.).

Allergen-specific immunotherapy (AIT) is the only etiology-based treatment capable of disease modification, as demonstrated by prevention of both the onset of new allergic sensitizations and disease progression.

Due to its disease-modifying effects based on its immunomodulatory properties, AIT is the only real curative modality in allergic asthma [10••].

Recent evidence has provided a plausible explanation for the multiple mechanisms of AIT, which induce both rapid desensitization and long-term allergen-specific immune tolerance, as well as the suppression of allergic inflammation in the affected tissues. The described mechanisms include changes in the profile of allergen-specific memory T- and B-cell responses, the synthesis of specific antibody isotypes that skew the immune response towards a non-inflammatory pattern, as well as decreased activation, tissue migration, and degranulation of effector cells including mast cells, basophils, and eosinophils [11]. These findings, together with the new biotechnological approaches, create a platform for development of the advanced vaccines. Moreover, reliable biomarkers could be selected and validated with the intention to select the patients who will benefit most from this immune-modifying treatment. Thus, AIT could provide a complete cure for a larger number of allergic patients [12].

## Treatment

AIT involves the repeated administration of allergen preparations in order to induce clinical and immunologic tolerance to the offending allergen. The two most commonly prescribed routes for AIT are subcutaneous (SCIT) and sublingual (SLIT). The sublingual route has emerged as an effective and safer alternative to subcutaneous administration. Factors considered for the selection of route include vaccine availability or approval, geographic location, cost, and the patient’s characteristics or the physician’s or patient’s preference [10••]. The quality of the allergen preparations used for AIT has been constantly improved. In addition, novel vaccines are being developed by using novel adjuvants, or changing the allergen to reduce allergenic activity, increase immunogenicity. Cloning of allergen proteins with use of recombinant DNA technology enabled the production of vaccines that have well defined molecular, immunologic, and biological characteristics. Genetic engineering enables modifications of the molecular structure of allergens [13].

Both SCIT and SLIT are of proven value in asthma. Numerous double-blind, placebo-controlled trials have confirmed that SLIT and SCIT are effective in reducing symptom scores and medication use, improving quality of life in asthma, and inducing favorable changes in specific immunologic markers [10••, 12]. Overall, moderate-to-high (somewhat weaker in children) evidence was found for the efficacy and safety of both SCIT and SLIT for the treatment of allergic asthma, and it has not been possible to assess superiority of either route over the other [14••, 15••].

However, the current documents of ARIA (Allergic Rhinitis and its Impact on Asthma) [16, 17] give both SCIT and SLIT a conditional recommendation in allergic asthma due to moderate and low quality of evidence, respectively. According to the GINA (Global Initiative for Asthma) report updated in 2012, AIT should be considered only after strict environmental avoidance and pharmacologic intervention, including ICS [17].The evidence for SCIT efficacy has been analyzed in the Cochrane review, which reported an overall clinical efficacy; that is, reductions in asthma symptom scores, medication usage, and allergen-specific bronchial hyperreactivity (BHR), and limited reduction in non-specific BHR [18]. The numbers of patients needed to treat in order to avoid asthma symptom deterioration or increase in medications were estimated as three and five, respectively. The effects on lung function were not consistent among trials. Also, more recent studies on efficacy of subcutaneous immunotherapy in asthma show similar treatment effect [19•, 20]. The most recent meta-analysis of the effectiveness of SCIT in the treatment of allergic rhinitis and asthma up to May 2013 concluded that SCIT reduces asthma symptoms and asthma medication usage. Respiratory adverse reactions to SCIT are common, but no deaths were reported in the included studies [21].

For the sublingual route of administration, most of the published evidence comes from studies primarily in rhinitis patients [22–26]. Thus, the studies are often not adequately powered for a definite conclusion. In addition, no consensus exists on the optimal endpoints. The efficacy of SLIT in seasonal allergy is now well documented both in adults and children. The data for perennial allergies is less convincing, particularly in children. In a large study including 602 asthmatic patients allergic to house dust mites (HDM), a reduced need for ICS for asthma control was demonstrated compared with placebo after only 1 year of treatment [27].

Recent systematic reviews graded the evidence for the effectiveness of SCIT and SLIT according to recommendations of the Grading of Recommendations Assessment, Development and Evaluation Working Group [14••]. In their review, Lin and colleagues point out that eight of 13 studies reported greater than 40 % improvement versus the comparator (placebo, pharmacotherapy, or other SLIT regimens) [Lin, #425, 15, 28].

In a review limited to a pediatric population receiving SCIT, SLIT, or usual care, it was concluded that SCIT reduces symptoms and medication scores, while SLIT can improve asthma symptoms [28••]. Another review including 74 references on SCIT and 60 on SLIT and eight comparative (SCIT versus SLIT) studies showed similar tendencies. A potential steroid-sparing effect of AIT is of utmost importance to avoid the potential adverse effects of ICS [14••].

In a recent study, it has been demonstrated that after 3 years of SLIT (birch pollen) in adult patients inadequately responding to a low dose of ICS, a significantly better control can be achieved by adding SLIT for 12 weeks [25]. In the ‘real-life’ retrospective study of Trebuchon et al., 63 % of patients with asthma due to sensitization to HDM who received SLIT showed improved symptoms and a reduction in medication. However, further studies specifically designed to address the effect of AIT in asthma are needed [29].

In particular, the ongoing phase III confirmatory, double-blind, placebo-controlled trials with both SCIT (Roxall Medizin, Allergopharma) and SLIT (ALK, Stallergen) in perennial HDM allergy will provide more solid evidence of the efficacy of AIT in asthma (data from ClinicalTrials.gov, EU Clinical Trials Register, Japan Pharmaceutical Information Center: Clinical Trials Information) [27].

### Contraindications and side effects

Along with general contraindications, severe or uncontrolled asthma is the most important and independent risk factor for both nonfatal and fatal adverse reactions to SCIT [10••].

A Cochrane systematic review [18] showed that the possibility of local or systemic adverse effects due to SCIT must be considered. If 16 patients are treated with SCIT, one would be expected to develop a local adverse reaction, and if nine patients are treated, one would be expected to develop a systemic reaction of any grade of severity. Thus, patients should be observed typically for 30–45 minutes after injection to assure proper management of systemic reactions. [30]. SLIT has been shown to be safer than SCIT and so far no fatalities have been reported. The side effects of SLIT are mainly local. In total, 11 cases of anaphylaxis were reported during SLIT but asthma was not considered a possible risk factor. Nevertheless, SLIT is not recommended to be administered in uncontrolled disease [31•].

### Standard dosage

#### Dosing

For many allergens, effective SLIT or SCIT doses have not been established. With grass pollen, the effective cumulative SLIT doses appear to be as high as 20 to 30 times greater than the effective SCIT doses.

Multiallergen SLIT has not been well studied, and its use might be limited by the increased cost of allergen extracts and the inconvenience of taking multiple doses.

The comparative effect of precoseasonal and continuous grass pollen SLIT in children has been investigated by Stelmach et al. [32] in a 2-year, prospective, randomized, double-blind, placebo-controlled trial. Both precoseasonal and continuous regimens were similarly associated with a substantial reduction in the combined symptoms/medication score, including the asthma score, when compared with placebo.

### Duration of treatment

The optimal duration for an AIT course is still a matter of debate, especially for SLIT. A recent study in asthmatic children showed that 3 years of SCIT is an adequate duration for the treatment of asthma in HDM-allergic subjects [19•].

### Cost/cost effectiveness

Studies comparing cost effectiveness between patients treated for 3 years with AIT versus those treated with pharmacotherapy alone have indicated that AIT might be associated with cost savings as high as 80 % 3 years after completion of treatment [10••].

### Emerging therapies

The most promising novel approaches with phase II and III clinical studies available or on the way include

- novel adjuvants (MPL, MAT technology)
- modified allergen molecules (further development of allergoids)
- peptides, recombinant allergens (birch, grass pollen, cat dander)
- new routes of AIT (e.g., intralymphatic, epicutaneous)

#### Pediatric considerations

No pediatric meta-analyses are available for SCIT. A more recent meta-analysis of SLIT in children reported a moderate effectiveness on asthma symptoms and medication intake [33]. However, a number of studies are characterized by shortcomings in sample size and methodology [34, 35]. New well controlled studies are postulated by the European Medicines Agency (EMA) within the Paediatric Investigation Plan (PIP).
